# Tumor microenvironment-driven non-cell-autonomous resistance to antineoplastic treatment

**DOI:** 10.1186/s12943-019-0992-4

**Published:** 2019-03-30

**Authors:** Yidi Qu, Bo Dou, Horyue Tan, Yibin Feng, Ning Wang, Di Wang

**Affiliations:** 1 School of Life Sciences, Jilin University, Changchun, 130012 China; 2 School of Chinese Medicine, The University of Hong Kong, Hong Kong, China

**Keywords:** Tumor, Non-cell-autonomous drug resistance, Tumor microenvironment, Drug resistance

## Abstract

Drug resistance is of great concern in cancer treatment because most effective drugs are limited by the development of resistance following some periods of therapeutic administration. The tumor microenvironment (TME), which includes various types of cells and extracellular components, mediates tumor progression and affects treatment efficacy. TME-mediated drug resistance is associated with tumor cells and their pericellular matrix. Noninherent-adaptive drug resistance refers to a non-cell-autonomous mechanism in which the resistance lies in the treatment process rather than genetic or epigenetic changes, and this mechanism is closely related to the TME. A new concept is therefore proposed in which tumor cell resistance to targeted therapy may be due to non-cell-autonomous mechanisms. However, knowledge of non-cell-autonomous mechanisms of resistance to different treatments is not comprehensive. In this review, we outlined TME factors and molecular events involved in the regulation of non-cell-autonomous resistance of cancer, summarized how the TME contributes to non-cell-autonomous drug resistance in different types of antineoplastic treatment, and discussed the novel strategies to investigate and overcome the non-cell-autonomous mechanism of cancer non-cell-autonomous resistance.

## Introduction

There has been spectacular advances and successes in the development and clinical application of small molecule antineoplastic drugs in the past several decades [1]. While cytotoxic compounds with more potent tumor-killing effects are still being discovered, molecularly targeted drugs are under development following the identification of promising targets in cancers [2]. Both cytotoxic chemotherapeutics and targeted treatments have significantly improved the survival of patients with cancers. As far, the majority of antineoplastic treatments are small-molecules, which have had great success in saving the lives of patients with cancer [3].

However, drug resistance is frequently developed during the clinical application of antineoplastic agents [4]. A substantial percentage of cancer patients exposed to an antineoplastic agent either does not benefit from the treatment (primary resistance) and show reduced responsiveness and undergo tumor relapse progression (secondary resistance) [5]. Although new compounds and combinations of drugs with higher potency in killing cancer cells have been developed, the nearly inevitable development of drug resistance has limited the clinical efficacy and effectiveness of antineoplastic treatment [6].

Both intrinsic and extrinsic biological causes of cancer drug resistance have been postulated. First, the overexpression of several transmembrane transporters in tumor cells, such as p-glycoproteins and multidrug resistance protein family members, reduces the intracellular drug concentration by restricting drug absorption and promoting drug efflux [7–9]. Second, changes in drug metabolism and drug targets, such as modifications of drug metabolizing enzymes by mutation and altered expression, lead to the dysregulation of prodrug activation and inactivation of the active form of the drug, thereby subsidizing the drug efficacy and promoting drug resistance [6, 10, 11]. Third, gene amplification in tumor cells increases the number of copies of oncogenes, which then reinforces oncogenic signaling during drug treatment [8]. Mutations in DNA repair systems might also promote resistance to antineoplastic agents by increasing DNA mutations and adapt to the drug [12, 13]. Fourth, pre-existing or acquired tumor cell heterogeneity might lead to variation in the response of cancer cells to antineoplastic agents [11]. For example, cancer stem cells, a subpopulation of cells that possess self-renewal and differentiation abilities, are more resistant to therapy than well-differentiated tumor cells [14].

**Fig. 1:**
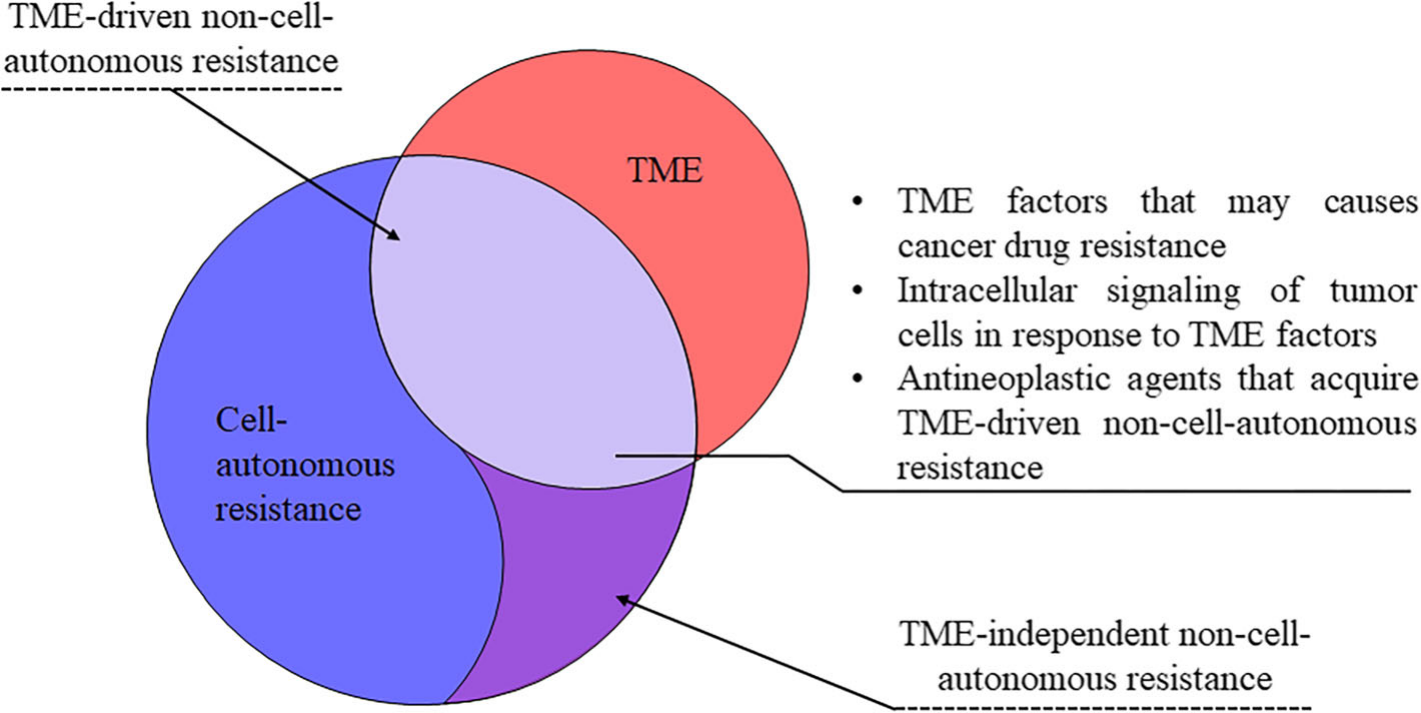
The role of the TME in the development of non–cell-autonomous resistance to antineoplastic agents

Although most of these mechanisms have been validated in patients, models of tumor cell-derived resistance have apparent limitations. Cancer cells typically interact with stromal cells within solid tumors in vivo, and these interactions extensively contribute to tumor development and therapeutic resistance. Thus, a new concept has been proposed in which tumor cells resistance to antineoplastic agents may be due to both cell-autonomous and non-cell-autonomous mechanisms. While the cell-autonomous mechanisms of cancer resistance have been reviewed elsewhere [6, 11], our knowledge of non-cell-autonomous mechanisms underlying tumor cell resistance to different treatments is incomplete. In particular, previous studies have highlighted the role of the tumor microenvironment (TME) in the development of non-cell-autonomous resistance to antineoplastic agents. Hence, in this review, we outlined the role of the TME in the development of non-cell-autonomous resistance to different antineoplastic agents. Intracellular signaling of tumor cells response to TME was discussed and how TME involved in resistance of each antineoplastic agent was depicted (Fig. 1).

## Non-cell-autonomous mechanisms of drug resistance in tumors

Cell-autonomous resistance, which is the “intrinsic” mechanism of resistance, involves the activation of alternative signaling pathways, acquisition of secondary mutations in drug targets, amplification of the target genes, and activation of efflux pumps. Extensive strategies to overcome cell-autonomous resistance have been developed, including but not limited to the development of new and more potent compounds, novel combined regimens of treatment and discovery of novel targets. Nonetheless, non-cell-autonomous mechanisms of resistance in tumors have only recently been highlighted, which suggests that drug failures and tumor relapse are closely related to factors in the surrounding TME [5].

**Fig. 2:**
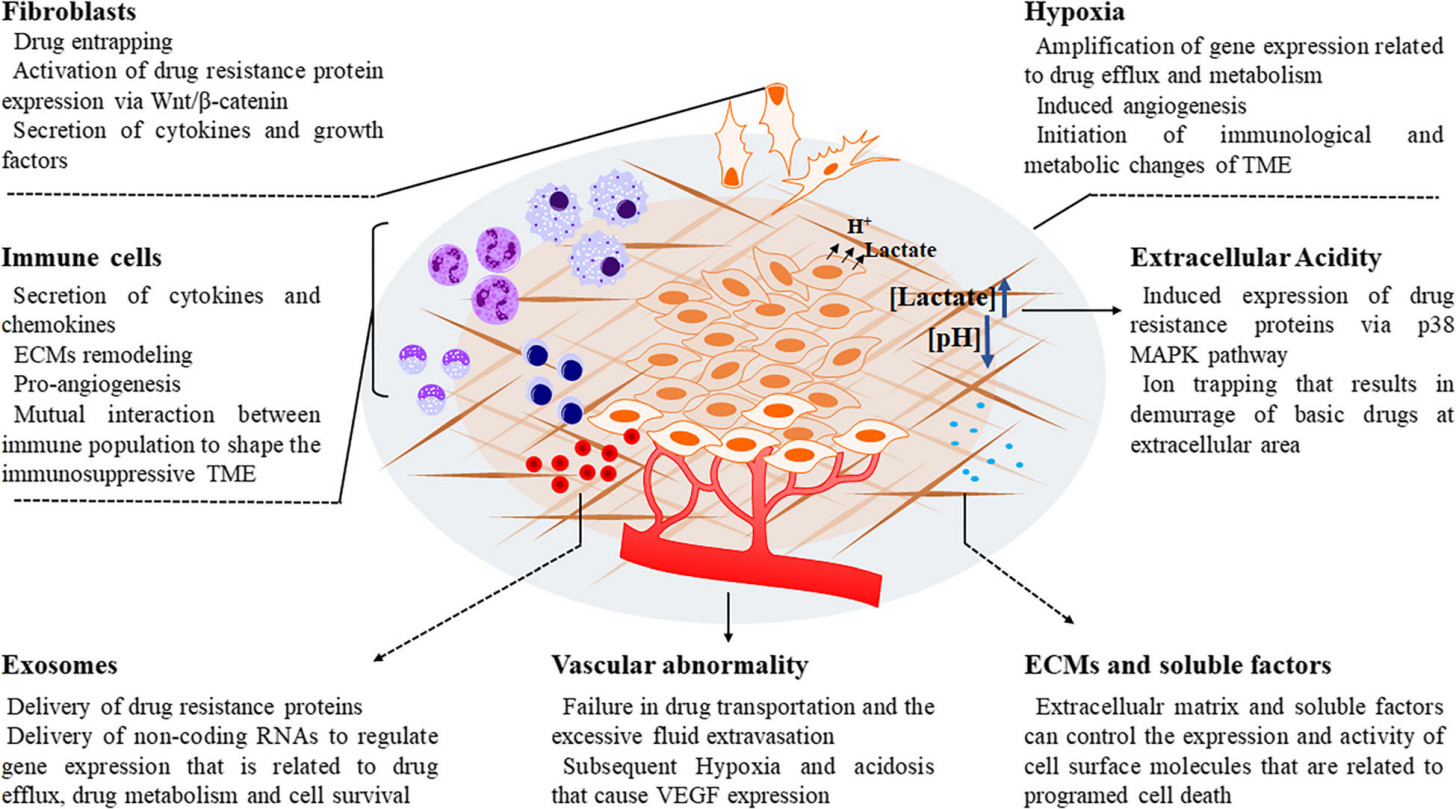
The main factors of tumor microenvironment-driven non-cell-autonomous drug resistance

Human tumors consist of both epithelial-like tumor cells and their surrounding cells and extracellular components, such as vasculature, fibroblasts, immune cells, endothelial cells and extracellular matrix (ECM). The surrounding components interact with tumor cells to form a microenvironment that favors tumor cell proliferation and survival [15]. The concept of the TME was introduced to illustrate that cancer progression is influenced by factors other than tumor cells. As a result, it was postulated that the TME might mediate the acquisition of resistance when tumors are exposed to antineoplastic agents in vivo [16–18]. Indeed, noninherent-adaptive drug resistance refers to non-cell-autonomous resistance, which relies on the treatment process rather than genetic or epigenetic changes and is closely related to the TME [19]. The TME may play a role in the initiation and maintenance of non-cell-autonomous drug resistance through various mechanisms, including hypoxia, extracellular acidity, vascular abnormalities, changes in immune populations, cancer-associated fibroblasts (CAFs) and their secretomes, exosomes, extracellular matrix, and other soluble factors. The overall regulatory mechanisms of non-cell-autonomous cancer resistance involving the TME are shown in Fig. 2 and have been reviewed in detail elsewhere [20, 21]. The mechanism involved in the non-cell-autonomous resistance to specific agents will be discussed later in this manuscript.

## Intracellular signaling in tumor cells in response to the TME

Upon changes in the microenvironment, tumor cells may initiate a series of intracellular signaling cascades to transfer these messages from the aforementioned environmental factors into cytoplasm and nuclei. Signal transduction between the TME and tumor cells may occur through direct cell-cell contact or binding of TME-derived ligands with their specific receptors on tumor cell surface. Intracellular signaling pathways are then altered to regulate the expression and activity of downstream effector molecules that confer drug resistance on tumor cells. These signaling pathways, including the mTOR, NF-κB, AKT, and STAT3 pathways, are generally associated with tumor cell proliferation and survival.

### mTOR signaling

As a vital signaling molecule that controls cell proliferation and growth under physiological conditions, mTOR is constitutively active in multiple types of cancer [19]. mTOR signaling is activated by the therapy-induced secretome, a collection of ambiguous components that form in TME after drug administration. Inhibition of mTOR signaling was found to blunt non-cell-autonomous resistance induced by vemurafenib, crizotinib or erlotinib (ERL) [22]. Lactate in the TME is imported by tumor cells and upregulates mTOR signaling via glutamine metabolism during treatment with vascular endothelial growth factor (VEGF) inhibitors. Activation of mTOR signaling initiates metabolic symbiosis in cancer cells, which confers resistance to VEGF inhibitors [23]. The switch towards the senescence-associated secretory phenotype (SASP) of tumor cells is activated by mTOR signaling and promotes non-cell-autonomous resistance. DNA damage-associated signaling through the ATM-TRAF6-TAK1 axis after initial drug treatment is responsible for the activation of mTOR signaling [24].

### NF-κB signaling

NF-κB signaling determines the expression of immunomodulatory and secretory factors, which are key factors for cell senescence in the non-cell-autonomous mechanisms of drug resistance [25]. Cell senescence is an antiproliferative program, and antineoplastic therapy often aims to limit cell proliferation [26]. NF-κB-driven IL-6 and IL-8 expression modulate the initiation and persistence of the SASP. In an in vivo model of lymphoma, the SASP is destroyed by NF-κB inhibition, leading to escape from immunosurveillance by natural killer cells and p53 inactivation, thereby producing drug resistance [27].

### AKT signaling

Both AKT and its associated signaling pathways are directly or indirectly involved in the oncogenic pathways in most human tumors [28]. Nevertheless, surprisingly results have been observed in which inhibition of AKT signaling in cancer cells confers drug resistance to the tumors. AKT-inhibited cancer cells activate their endomembrane system in a posttranscriptional manner to secrete inflammatory proteins IL-6 and IL-8, and extracellular vesicles (EVs), which enable rapidly growing cells to better withstand the stress conditions induced by drug treatment. These data suggest that AKT inhibition may contribute to the non-cell-autonomous mechanism of drug resistance in vivo [29].

### STAT3 signaling

STAT3 signaling is generally considered the pathway in tumor cells that responds to the inflammatory TME [30]. A series of cytokines and chemokines can activate STAT3 in tumor cells, among which IL-6 and IL-1β are the major inducers derived from the TME. Tumor-associated macrophages (TAMs), neutrophils and CAFs in the TME are the major cell types that secrete IL-6 and IL-1β and are responsible for the activation of STAT3 in tumor cells [31]. Several downstream effectors of STAT3 in tumor cells were reported to contribute to non-cell-autonomous resistance to antineoplastic treatment. STAT3 activation may confer drug resistance by initiating epithelial-mesenchymal transition (EMT), suppressing epigenetic tumor suppressor microRNAs (miRNAs) and enhancing the expression of antiapoptotic proteins [32–34]. STAT3 activation in the TME may trigger vascular abnormalities, which were shown to abolish the proper delivery of gemcitabine [35]. STAT3 in tumor cells may also enhance the expression of Rab family proteins to facilitate exosome release, which confers cisplatin resistance in ovarian cancer [36].

## Antineoplastic drug resistance involving TME-driven non-cell-autonomous mechanisms

TME-driven non-cell-autonomous mechanisms of resistance to various types of antineoplastic agents have been extensively studied. Individual drug may involve unique TME-driven non-cell-autonomous mechanisms, and resistance may come from more than one non-cell-autonomous factor in the TME (Table 1).

**Table 1:** A list of drugs being resistant in tumors by non-cell-autonomous methods

Drug	Tumor with resistance	Cancer cell lines	Experimental objects	Non-cell- autonomous factors	Signaling pathway	Reference
Bevacizumab	Ovarian cancer, GBM	SKOV-3, OVCAR-3, GOC-2, GOC-A2, U87-MG, U251, LN229,	Cell, xenograft tumor	UVEC, TAM	AKT/FGF2, MIF	[88, 125]
Bortezomib	Multiple myeloma (MM) , MCL	RPMI-8226, U266, 5T33MM mouse primary tumor cell	Cell, xenograft tumor, clinical sample	BMSC, TAM	MUC-1, IL-6, JAK2/STAT3, NF-κB	[88, 89, 146–149]
Cetuximab	Head and neck squamous cell carcinomas (HNSCC), lung squamous cell carcinoma, gastric cancer, NSCLC	UTSCC cell lines, EBC1, GTL16, HCC827	Cell, xenograft tumor	CAF	MMP family, HGF/MET, NF-κB	[107]
Cytarabine	Acute myelogenous leukemia (AML)	OCI-AML cell lines, Molm13, KG-1, HL-60, ML-1, Mono-Mac-6, Kasumi-1, NB-4, MV-4/11, HEL, CMK, M-07e, primary AML cell	Cell, xenograft tumor	MSC, hypoxia	Autophagy, FLT3/PI3K, Mcl-1	[63, 64, 150]
Doxorubicin	MM, osteosarcoma, aggressive N-Myc amplified neuroblastoma, melanoma, breast cancer, pancreatic cancer, NSCLC, colon cancer, soft tissue sarcoma, prostate carcinoma	NCI-H460, RH460, K562, K562Dox, MCF-7, Saos-2, U2-OS, U266, RPMI-8226, DP42, MM1.S, NCI-H929, 38ATLN, MDA-MB-231, CRL-2539, A375, U251, HCT116, HEK293T, BxPC-3, AsPC-1, A549, H2170, KLN205, CT26, WT-CLS1, SH-SY5Y, SK-N-BE2, SKLMS-1, RD, LNCaP, 22Rv1	Cell, xenograft tumor, clinical sample	ECM, CAF, MSC, hypoxia, MDSC	IL-6/STAT3, NF-κB/ IκB, IL1β, CXCL1/GROα, PI3K/Rac, VEGF, HIF-1α/miR424/PDCD4, cathepsin family, hyaluronic acid (HA), Rho/ROCK, miRNA/PTEN	[69, 70, 76–78]
Epirubicin	Breast cancer	MCF-7 and MDA-MB-468	Cell	CAF	Estrogen/GPER/cAMP/PKA/CREB	[84]
Erlotinib	Cholangiocarcinoma (CCA)	HuCC-T1, EGI-1, SK-ChA-1, Mz-ChA-1	Cell, xenograft tumor, clinical sample	CAF	IR/IGF2/IGF1R	[111]
Etoposide	Hepatoma, lung carcinoma, prostate carcinoma, melanoma, breast cancer	HepG2, A549, PC3MLN4, A375SM, MCF-7, RPMI-8226, MDA-MB-231, SUM-159PT	Cell, xenograft tumor	Hypoxia, myeloma cell	p53, acylglycerol-3-phosphate acyltransferase 2, p27/kip1,	[92, 93]
Fludarabine	Chronic lymphocytic leukemia (CLL)	NOX-A12, Jurkat cell, Nalm6,	Cell, clinical sample	BMSC, CLL B-cell, MSC	CXCL12/CXCR4, Akt/FoxO3a/Bim, IL-8, CCL4, CCL11, CXCL10, GSH	[151]
Gefitinib	Non-small cell lung cancer (NSCLC), breast cancer	SUM102, SUM149, PC-9, HCC827	Cell	CAF, hypoxia	FGF2, HGF/Met, IGF1/HIF-1α, podoplanin	[109, 110, 152, 153]
Gemcitabine	Pancreatic cancer, thymoma, lung cancer, melanoma, breast cancer	AsPC1, BxPC3, Panc1, MIAPaCa2, CFPAC1, HPAC1, THP1, K989, Capan2, SW1990, NIH-3T3, THP1, EL4, LLC, B16F10, 4T1	Cell, xenograft tumor, clinical sample	CAF, TAM, MDSC	Tissue transglutaminase /SATB-1/SDF-1/CXCR4, miR-365/CDA, IL-17, IL-1β	[57, 58, 154–157]
Ibrutinib	mantle cell lymphoma (MCL), CLL	HBL-2, Jeko-1, Mino, SP49	Cell, xenograft tumor, clinical sample	BMSC, nurse-like cell	PI3K/AKT/mTOR, integrin-β1, NF-κB, Bcl-2	[151]
Imatinib	Chronic myelogenous leukemia (CML)	K562, KU812		BMSC	NF-κB/STAT5, HO-1, PI3K/AKT, Bcl-2, CXCL12/CXCR4	[151]
Lapatinib	Esophageal squamous-cell carcinoma (ESCC), breast cancer	TE cell lines, EC-GI-10, KYSE cell lines, T.T, TTN, MCF10, MDA-MB-453, HCC1954, MCF7, T47D, SUM cell lines	Cell, xenograft tumor	CAF, ECM	HGF/Met, FGF/FGFR, Bcl-2/Bcl-x, PI3K/AKT, JAK/STAT, laminin/Bcl-2	[101–103, 158–160]
Mitoxantrone	Prostate cancer	Prostate cancer cell lines M12, 22Rv1, M2205, PC-3, DU145, LNCaP, PSC27, Hs5 and Hs27a	Cell, clinical sample	CAF,	Apoptosis	[83]
Oxaliplatin	Colorectal cancer	human colorectal cancer cells HCT 116 and SW620,	Cell	CAF, hypoxia	Nrf2, HIF	[37, 38, 40]
Cisplatin	Esophageal squamous cell carcinoma, Ovarian cancer, epithelial ovarian cancer	MCF-7, BC-MSCs, A2780 ovarian cancer cell lines	Cell	CAF, miRNA	AKT, ERK1/2, IL-6, STAT3, p38, JNK, STAT3, NF-κB	[36, 38–48]
Paclitaxel	Breast cancer, renal cell carcinoma (RCC)	MMTV-PyMT mouse primary tumor cell lines, MDA-MB-231	Cell, primary tumor, xenograft tumor	TAM	Cathepsin B and S	[90, 91]
Sorafenib	Hepatocellular carcinoma (HCC), prostate cancer, AML, thyroid carcinoma	22Rv1, PC-3, HCA-1, JHH-7, Hep3B, Huh7, PLC/PRF/5, HepG2, MHCC97H, HCCLM3, Hepa1-6, H22, Molm13, SMMC-7721, KTC1, TPC1	Cell, xenograft tumor, primary tumor, clinical sample	TAN, CAF, HSC, hypoxia, pericyte	SDF1α/CXCR4, Bcl-2, HIF-1α/NF-κB/CXCL5, CCL2&CCL17, BMX/STAT5, HIF-1α/hydroxyproline, collagen I, TGF-β1/CTGF, TSP- 1/TGFβ1, ERK, AKT, SMAD3	[112–119, 161, 162]
Sunitinib	RCC, GBM, breast cancer, colon cancer, melanoma, lymphomas, lung carcinoma	U87MG, 4T1, CT26, RENCA, 786-O, ACHN, 771R-Rluc, EL4, LLC, B16F1, TIB6	Cell, xenograft tumor, clinical sample	MDSC, macrophage	STAT5/IFN-γ	[90, 91]
Temozolomide	Glioblastoma (GBM)	U251, U87, GBM8401, U87MG, HEK293T, A172	Cell, xenograft tumor	Hypoxia, perivascular cell, astrocyte	ROS, HIF-1α, NF-κB, Bcl-x, miR-26a/Bad/Bax, MGMT, EGFR, PI3K/AKT, Ras/Raf, connexin43,	[51–54, 163–165]
Trastuzumab	Breast cancer	BT-474, SK-BR-3, MDA-MB-453, MDA-MB-361	Cell, xenograft tumor	Adipocyte	IFN-γ	[100]
Vatalanib	GBM	PDGC23, U87MG	Cell, primary tumor	Myeloid cell	Colony stimulating factor-1	[90, 91]
Vemurafenib	Melanoma, thyroid carcinoma	A375M6, WM266-4, M21, SkMel28, FS cell lines, M93-047, UACC cell lines, WM cell lines, 1205LU, YUMM1.7, KTC1, TPC1	Cell, xenograft tumor	Acidosis, CAF in aged TME, pericyte	mTOR, AKT, sFRP2/ROS, TSP- 1/TGFβ1, ERK, SMAD3	[121, 122]

### DNA-targeted drugs

#### Platinum-based chemotherapy

Platinum-based chemotherapy, including cisplatin, carboplatin, oxaliplatin, and nedaplatin, is the front-line treatment for several advanced cancers; however, treatment failure due to chemoresistance is common. In addition to the autonomous mechanism of resistance, such as the aberrant expression of antiapoptotic proteins in resistant tumor cells, some non-cell-autonomous resistances mechanisms could be involved. In colorectal cancer treatment, there is the possibility of drug resistance and tumor recurrence in patients treated with oxaliplatin, and the reason underlying this risk may be the changes in CAFs [37]. Oxaliplatin-based chemotherapy can increase hypoxia and the accumulation of CAFs in the TME, and hypoxia-inducible factor (HIF) activation. Moreover, expression of fibroblast growth factor 2 (FGF-2) increases significantly and initiates cancer proliferation and tumor vascular angiogenesis [38]. In esophageal squamous cell carcinoma, cisplatin treatment can promote PAI-1 secretion by CAFs, which acts in a paracrine manner to maintain AKT and ERK1/2 signaling in cancer cells and to promote cell survival [39]. These changes in cytokines are related to the accumulation of CAFs and mediate the mechanism of drug resistance [38, 40].

Conditioned media from ovarian cancer-associated mesenchymal stem cells (MSCs) was found to protect tumor cells by inhibiting endogenous proapoptotic signalings, such as that by XIAP and the caspases cascade [41]. Coculture of breast cancer cells with tumor tissue-derived MSCs (BC-MSCs) led to the development of cisplatin resistance; this process could be associated with the IL-6 secreted by BC-MSCs, which activates STAT3 signaling in breast cancer cells and promotes cell survival [42]. Another study suggested that the upregulation of IL-6 in MSCs could be dependent on cisplatin treatment [43].

EVs may also contribute to cisplatin resistance. Samuel et al. collected EVs from ovarian cancer cells and showed that they could activate the p38 and JNK pathways in bystander tumor cells. EVs uptake promoted in vitro resistance to cisplatin in ovarian cancer cells [44]. Further, it was found that hypoxia triggered ovarian cancer cells to secrete more exosomes, which in turn ameliorated dsDNA damage in cisplatin-treated cells and promote cell survival by activating the STAT3 pathway [36]. In breast cancer cells, exosomal miRNAs, such as miR-222/223, were found to play a role in facilitating the adaptation to a quiescent state during carboplatin-based therapy [45].

Some soluble stromal factors may also contribute to resistance to platinum-based chemotherapy. In epithelial ovarian cancer patients, stromal expression of periostin was associated with cisplatin resistance and clinical treatment outcomes. An in vitro study revealed that periostin caused persistent activation of AKT in A2780 ovarian cancer cells, leading to cell survival under cisplatin treatment [46]. Fibroblast activation protein alpha (FAP) expression in the stroma of epithelial ovarian cancer predicted the poor outcome of patients treated with cisplatin. Ovarian cancer cells treated in vitro with FAP showed significantly improved cell survival when exposed to cisplatin [47]. Platinum compounds may also trigger the ability of tumor cells to shape the immunosuppressive microenvironment, such as inducing M2 polarization of macrophages through the IL-6/STAT3 and NF-κB pathways; these changes indirectly contribute to the chemoresistance of cervical and ovarian cancers [48]. M2 macrophages may also produce nitric oxide to counteract the cisplatin-induced activation of syntaxin 4 and acid sphingomyelinase, thereby conferring tumor cells with chemoresistance [49].

#### Other alkylating agents

Acquired resistance to temozolomide (TMZ) has been reported in glioblastoma multiforme (GBM). In addition to resistance based on the modulation of DNA repair protein O6-methylguanine-DNA methyltransferase (MGMT) [50], the hypoxic TME was recently shown to affect drug sensitivity considerably. Cycling hypoxia was found to induce TMZ resistance in GBM, which was associated with ROS-mediated activation of HIF-1α and NF-κB, resulting in increased expression of the antiapoptotic protein Bcl-xL in GBM cell lines and xenograft tumors [51]. Furthermore, the hypoxic microenvironment could inhibit mitochondrial apoptosis by the HIF-1α-associated induction of miR-26a expression, which directly targets and suppresses proapoptotic Bad and Bax expression to protect mitochondrial function [52]. Besides, non-cell-autonomous resistance to TMZ may stem from factors in the surrounding environment. The perivascular niche that comprises endothelial and stromal cells was shown to support the resistance of GBM cells to TMZ treatment. Coculture of GBM cells with perivascular niche cells led to the activation of MGMT, epidermal growth factor receptor (EGFR), PI3K/AKT and Ras/Raf signaling that promoted TMZ resistance [53]. Direct contact between astrocytes and GBM cells through connexin43-dependent gap junctional communication might protect tumor cells from TMZ-induced apoptosis [54].

#### Nucleotide analogs and precursor analogs

Gemcitabine resistance is commonly reported in pancreatic cancer. Previous studies have shown that the resistance mechanism may involve non-cell-autonomous changes in signaling pathways within tumor cells due to contact with CAFs [55, 56]. In pancreatic ductal adenocarcinoma, CAFs are activated in response to tissue transglutaminase in the TME and initiate signaling pathways in tumor cells related to gemcitabine resistance. This process could be attributed to the overexpression of SATB-1 in tumor cells near stimulated CAFs, which then upregulate the secretion of SDF-1, a cytokine that plays a crucial role in many types of solid tumors by initiating signaling through its receptor CXCR4 [57, 58]. Another study suggested that gemcitabine resistance may be associated with TAM-derived exosome and exosomal miRNAs. Exosomal miR-365 was identified as the critical mediator of gemcitabine resistance in pancreatic ductal adenocarcinoma; it modulates pyrimidine metabolism and upregulates CDA expression, which inactivates gemcitabine by conversion to dFdUridine [59]. Furthermore, gemcitabine was found to induce inflammasome activation and IL-1β production in myeloid-derived suppressor cells (MDSCs), which in turn induced IL-17 secretion by CD4^+^ T cells to blunt gemcitabine toxicity [60].

Cytarabine is used for the treatment of acute myelogenous leukemia (AML). A previous study showed that cytarabine resistance might arise from intercellular communication between AML and bone marrow-derived MSCs [61]. AML cells cocultured with MSCs had elevated levels of Mcl-1, which is associated with multidrug resistance [62], and AML cell autophagy induced by MSC conferred cytarabine resistance [63]. In addition, the hypoxic microenvironment downregulated FLT3 expression in AML cells, which was associated with suppression of the PI3K pathway. Reduced FLT3 expression led to a lack of response to cytarabine treatment [64].

Fludarabine is used for the treatment of chronic lymphocytic leukemia (CLL). Bone marrow stromal cells (BMSCs) may create a CXCL12 gradient to promote the migration of CLL B cells, which promotes fludarabine resistance in CLL [65]. This response could be attributed to the binding of environmental CXCL12 to CXCR4 on CLL B cells and the subsequent activation of the AKT/FoxO3a/Bim axis within tumor cells [66]. Trimaco et al. also proved that MSCs isolated from the bone marrow of CLL patients rendered CLL B cells resistant to fludarabine in coculture conditions, which could be related to the presence of cytoprotective cytokines such as IL-8, CCL4, CCL11, and CXCL10 [67]. Furthermore, BMSCs could induce an increased import of cystine and its conversion into cysteine in the TME, and the resulting cysteine could be taken up by CLL B-cells for GSH synthesis. The intracellular redox balance maintained by GSH protects CLL cells from fludarabine toxicity [68].

### Cytotoxic drugs

#### Anthracyclines

Doxorubicin/Adriamycin is an anthracycline widely used to treat various types of cancer, and doxorubicin resistance is frequently observed and involves a non-cell-autonomous mechanism. A study by Tu and colleagues showed that in vitro and in vivo interactions between MSCs and the osteosarcoma cell lines Saos-2 and U2-OS led to doxorubicin resistance through intercellular signal transduction involving the IL-6/STAT3 axis. MSC-derived IL-6 protects tumor cells from doxorubicin-induced apoptosis by activating STAT3 signaling [69]. The intercellular activation of survival signals by MSCs was also observed in multiple myeloma (MM), in which MSCs initiated NF-κB signaling through autophagy-dependent IκB degradation in MM cells [70].

MDSCs of a particular phenotype, with a polymorphonuclear structure and neutrophils in bone marrow were reported to mediate doxorubicin resistance through the secretion of soluble factors [71] including IL-1β, which was shown to activate PI3K/Rac and IL-1RI/β-catenin-dependent BIRC3 transcription in breast cancer cells, and CXCL1/GROα which increased angiogenesis in a mouse model of breast cancer [72, 73].

Zhang and colleagues suggested that the endothelial cell population may play a role in doxorubicin resistance in soft tissue sarcoma by facilitating vascular abnormalities. The overexpression of VEGF induces doxorubicin resistance without overtly impacting tumor cells but promotes endothelial cell proliferation, migration ,and sensitivity to doxorubicin. The addition of an anti-VEGF monoclonal antibody significantly improved doxorubicin sensitivity in soft tissue sarcoma [74].

Coculture of prostate cancer cells with CAFs attenuated doxorubicin-induced DNA damage and cytotoxicity. This effect of CAFs was attributed to the blockade of doxorubicin accumulation in prostate cancer cells due to increased cancer cell glutathione levels, which inhibited doxorubicin-induced ROS production [75].

Besides, non-cell factors in the TME also contribute to non-cell-autonomous doxorubicin resistance. The hypoxia-mediated induction of miR-424 in tumor cells promotes doxorubicin resistance. The HIF-1α-binding sequence in A375 melanoma cells, U251 glioblastoma cells, HCT116 colon cancer cells, A375 cell xenografts, and clinical breast cancer samples directly increased the transcription of miR-424, which suppressed the levels of the apoptosis-associated factor PDCD4 and protected cells from apoptosis [76]. In addition, the accumulation of hyaluronic acid (HA) in the TME plays an essential role in maintaining hypoxia which was shown by TME remodeling in many types of cancer cell lines [77]. In aggressive N-Myc-amplified neuroblastoma cells, the cathepsin family in the ECM contributes to doxorubicin-resistance [78]. Joyce et al. suggested that ECM changes in the breast cancer microenvironment, such as increased stiffness, led to the nuclear translocation of YAP in MDA-MB-231 cells. The subsequent mesenchymal differentiation contributed to ECM-induced doxorubicin resistance in breast cancer [79]. Ebata et al. showed that Rho/ROCK-associated myosin activation was also involved in ECM stiffness-induced doxorubicin resistance in MCF-7 breast cancer cells [80]. Also, microvesicle-like EVs were shown to carry drug efflux pump proteins from resistant Chronic myelogenous leukemia (CML) cells to sensitive tumor cells, which consequently reduced the intracellular availability of doxorubicin [81]. Resistant breast tumor cell-derived exosomes contain several miRNAs, such as miR-100, miR-17, miR-222, miR-342p and miR-451, among which miR-222 suppressed PTEN expression in recipient drug-sensitive cells to gain resistance to doxorubicin [82].

CAFs seem to play a role in the resistance to other anthracyclines. Genotoxic stress can induce DNA damage in prostate cancer stromal fibroblasts that leads to the expression and secretion of a glial cell-derived neurotrophic factor, which has a paracrine effect on prostate tumor cells resulting in acquired resistance to mitoxantrone [83]. In breast cancer, CAFs activate a novel estrogen/GPER/cAMP/PKA/CREB signaling axis that triggers the switch to aerobic glycolysis, and the production of extra pyruvate and lactate enables tumor cells to survive epirubicin treatment [84].

As anthracyclines are alkaline chemotherapeutic agents, they tend to have limited absorption in the acidic TME. This is due to a phenomenon called ion trapping, which refers to the preference of alkaline chemotherapeutic agents to accumulate in areas of low pH. As tumor cells tend to maintain a neutral pH by overexpressing proton pumps protein, the extracellular pH is more acidic [85, 86]. This pH gradient hinders the absorption of alkaline chemotherapeutic drugs by tumor cells and prevents the chemicals from reaching their site of action [87].

#### Other cytotoxic drugs

Bortezomib (BTZ), the first proteasome inhibitor approved for clinical use, was usually applied in the treatment of MM and mantle cell lymphoma (MCL). Coculture of MM cells with BMSCs was recently shown to induce BTZ resistance, which was associated with the aberrant expression of MUC-1, a vital factor for BTZ-resistance in MM. Further observation suggested that IL-6 secreted from BMSC upregulated MUC-1 via the JAK2/STAT3 pathway in MM cells [88]. TAMs also participated in the mechanism of BTZ resistance. A study by De Beule and colleagues showed that TAMs might activate STAT3 signaling and reduce the apoptosis of MM cells through the JAK2 pathway upon BTZ treatment. In vivo co-treatment with BTZ and an ATP-competitive JAK2 inhibitor improved the drug sensitivity of MM [89]. Overall, it can be concluded that the STAT3 pathway plays a vital role in BTZ-resistance in MM.

Paclitaxel, also known as Taxol, can interfere with the normal function of microtubules during tumor cell division. The mechanism of paclitaxel resistance may also be associated with TAMs. The Taxol-treated MMTV-PyMT mouse breast cancer model showed the increased abundance of TAMs, which expressed and released proteases such as cathepsins B and S to prevent tumor cells from undergoing Taxol-induced cell death. This protective effect of TAMs on breast ductal carcinoma cells was independent of direct cell-cell contact [90, 91].

Hypoxia is the major TME factor that promotes non-cell-autonomous resistance to etoposide. In HepG2 cells, hypoxia induced a reduction in p53 to protect cells from etoposide-induced apoptosis and promoted the DNA binding activity of c-jun to prevent DNA damage [92, 93]. Interestingly, Dudley et al. showed that tumor stromal cells are less sensitive to etoposide-induced p53 activation, which endows prostate cancer with drug resistance [94]. The expression of acylglycerol-3-phosphate acyltransferase 2 (AGPAT2) under hypoxic conditions increases lipid droplet accumulation in multiple types of cancer cells, leading to etoposide resistance [95]. Besides, non-cell-autonomous mechanisms of etoposide resistance seem to involve cell adhesion. The adhesion of myeloma cells to fibronectin led to G0/G1 cell cycle arrest, which depends on increased p27/kip1 protein levels and the inhibition of Cyclin A-and Cyclin E-associated kinase activity. Disrupting the interaction between fibronectin and tumor cells initiated cell cycle progression into S phase, which reverted MM cells to an etoposide-sensitive phenotype [96].

### Tyrosine kinase inhibitors (TKIs)

#### Human epidermal growth factor receptor (HER2) inhibitors

Overexpression of HER2 plays a crucial role in cancer development due to its function in stimulating cell growth and differentiation. HER2 inhibitors, including monoclonal antibodies and small-molecule TKIs, have been developed for the treatment of diverse types of cancer, especially breast cancer [97–99]. Recent studies have shown that TME-driven non-cell-autonomous mechanisms are involved in resistance to anti-HER2 treatment. Breast tumors next to adipose tissue were found to be more resistant to trastuzumab treatment, which could be associated with the adipose tissue-induced failure of antibody-dependent cellular cytotoxicity. Adipocytes reduce the secretion of interferon-γ (IFNγ) by natural killer cells and induced expression of survival genes in breast tumor cells, leading to trastuzumab treatment failure [100]. Acquired resistance to another anti-HER2 therapy lapatinib was demonstrated in esophageal squamous-cell carcinoma cell lines; this resistance could be associated with CAFs-secreted molecules, including HGF and FGF, which activate the HGF/Met and FGF/FGFR pathways to induce significant resistance to lapatinib [101]. Another study suggested that the spatial proximity of breast ductal carcinoma cells to CAFs also influences lapatinib resistance, as the induction of antiapoptotic Bcl-2/Bcl-x, PI3K/AKT ,and JAK/STAT signaling was observed in lapatinib-treated tumor cells, and this induction was associated with CAF-induced protection by HA in the stroma and with intercellular communication between tumor cells and CAFs through JAK/STAT signaling [102]. In addition, ECM components such as laminin may affect breast ductal carcinoma sensitivity to lapatinib. Tumor cells in niches with laminin-enriched ECM express more antiapoptotic Bcl-2 family proteins and exhibited resistance to lapatinib [103]. These previous studies suggest that multiple non-cell-autonomous mechanisms may be involved in the resistance of tumor cells to anti-HER2 treatment.

#### EGFR inhibitors

EGFR inhibitors are compounds and antibodies that suppress the activity of either wild-type or mutant EGFR and downstream signaling. As an essential growth pathway, EGFR signaling is generally hyperactive in various types of human cancer [104]. Acquired resistance to cetuximab (CTX), a monoclonal antibody that can block the binding of EGF to EGFR and inhibit the activation of downstream pathways AKT and ERK1/2, was found in head and neck squamous cell carcinomas (HNSCC) [105, 106]. Coculture of HNSCC cells with CAFs significantly reduced CTX-induced growth inhibition, which may be associated with increased expression of MMP-1 in both HNSCC cells and CAFs. The elevation of MMP-1 was due to CAF-derived soluble factors, and MMP-1 can cooperate with other MMPs in the ECM to protect tumor cells from CTX-induced growth inhibition [107]. Another study suggested that CAFs produce HGF in an NF-κB-dependent manner and HGF activates Met-dependent signaling in non-small cell lung cancer. The environmental level of lactic acid promoted HGF production by CAFs and acquired resistance to EGFR TKIs [108].

Gefitinib (GFT) and ERL are EGFR TKIs that are usually used to treat non–small cell lung cancer. It was recently shown that most of the non-cell-autonomous mechanisms of GFT and ERL resistance involve the action of CAFs. The HGF/Met signaling pathway in CAFs is involved in GFT resistance in triple-negative breast cancer, and secreted HGF confers resistance by increasing Met phosphorylation in breast cancer cells [109]. A specific population of CAFs expressing podoplanin was found to be associated with GFT resistance. Patients with higher populations of podoplanin-positive CAFs exhibit worse outcomes after GFT treatment; this finding was supported by the observation of increased ERK1/2 pathway activity in GFT-treated cancer cells cocultured with podoplanin-positive CAFs [110]. In cholangiocarcinoma (CCA), the interaction between cancer cells and CAFs mediated by insulin-like growth factor 2 (IGF2), insulin receptor (IR) and IGF1 receptor (IGF1R) was found to regulate ERL resistance. IGF2 expression in activated CAFs initiates IR/IGF1R-mediated proliferation and survival signaling in cancer cells and induces the production of more IGF2 as a positive feedback to promote CAFs proliferation when CCA tumors are exposed to ERL, leading to an adaptive mechanism by which CCA tumors escape death by ERL treatment [111].

#### B-Raf inhibitors

B-Raf belongs to the Raf family of kinases and is frequently mutated and hyperactive in multiple types of cancers to facilitate uncontrolled cell growth. The B-Raf V600E mutant inhibitor sorafenib was recently developed to treat liver and renal cancers. The involvement of TME factors, including CAFs, TAMs and tumor-associated neutrophils (TANs), was extensively reported in recent studies. Coculture of CAFs with prostate cancer cells induces sorafenib resistance, which can be overcome by a Bcl-2 inhibitor [112]. In hepatocellular carcinoma (HCC), sorafenib treatment induces SDF1α expression in the stroma, which in turn activates hepatic stellate cells (HSCs) and Gr^+^ myeloid cell infiltration through a CXCR4-dependent pathway. Sorafenib-activated CXCR4 signaling may contribute to the resistance mechanism by inducing the infiltration of TAMs and regulatory T cells into the TME [113, 114]. In particular, the activation of HSCs induces collagen I and transforming growth factor-β (TGF-β) expression, which increases cell-cell contacts in spheroid culture to cause resistance to sorafenib and cisplatin in HCC cells by regulating EMT activation [115, 116]. It was also reported that TANs affect the sensitivity of HCC to sorafenib. The transformation from normal neutrophils into TANs is a response to the sorafenib-induced hypoxic microenvironment in HCC, which activates the HIF-1α/NF-κB pathway to promote CXCL5 expression. Hypoxia was shown to inhibit TAN apoptosis. As a result, TANs secrete CCL2 and CCL17 to recruit inflammatory macrophages and Treg cells, and cancer tissues in an environment with these cells have a substantially increased tumor microvascular density [117]. Another study with AML showed that sorafenib treatment could induce hypoxia, which evoked the upregulation of Tec family kinase (BMX) expression in AML cells, leading to the activation of STAT5-dependent signaling associated with resistance [118]. Another hypoxia-associated mechanism involves metabolic perturbations that are relevant to the initiation of HCC resistance by hydroxyproline augmentation and accumulation. Under hypoxic conditions, hydroxyproline is tightly related to HIF-dependent tumor phenotypes and glutamine-proline conversion in both normal and tumor cells and confers sorafenib resistance in HCC [119]. It was discovered that HSCs are triggered to myofibroblast-like cells by HCC, and enhanced collagen I expression results in sorafenib resistance and HCC cell migration [115]. This is probably due to the expression of TGF-β1 and CTGF in coculture of HSCs and HCC, which promotes an EMT-like transformation and a collective migration [116].

Vemurafenib was developed to target V600E mutant B-Raf in melanoma. Studies have shown that multiple mechanisms are involved in the acquisition of non-cell-autonomous resistance to vemurafenib [120]. The acidosis in TME was reported to promote the constant phosphorylation of AKT in BRAF-mutated melanoma cells, which activates mTOR signaling and confers vemurafenib resistance [121]. The mature microenvironment increases oxidative stress to augments vemurafenib resistance through a factor secreted by CAFs, sFRP2. As a β-catenin inhibitor, sFRP2 suppresses the production of APE1 via MITF inactivation, losing control of ROS reactions [122].

Besides, there are reports of resistance in thyroid cancer to the combination of sorafenib and vemurafenib, which was associated with the presence of pericytes in the TME. B-Raf inhibitor-treated pericytes secrete TSP-1 to activate the TGFβ1 axis and thus recover ERK, AKT and SMAD3 pathway activity in tumor cells, leading to increased survival and cell death refractoriness [123].

#### VEGF/VEGFR inhibitors

Bevacizumab is a humanized monoclonal antibody against VEGF-A that blocks angiogenesis in tumors. In ovarian cancer, mutual cross-talk between tumor cells and umbilical vein endothelial cells (UVECs) activates AKT-associated signals in both cell types, thereby inducing the secretion of FGF-2 by HUVECs. The activation of AKT and the secretion of FGF2 were shown to contribute to bevacizumab resistance [124]. In GBM cells, bevacizumab seems to enhance the recruitment of myeloid macrophages, which tended to polarize towards an immunosuppressive M2 phenotype. This could be attributed to the downregulation of macrophage migration inhibitory factor (MIF) in GBM upon VEGF inhibition by bevacizumab [125].

Sunitinib is a VEGFR inhibitor that blocks angiogenesis in multiple types of cancer. The exosome-derived lncRNA LNCARSR sponges tumor suppressive miR-34 and miR-449 to encourage sunitinib resistance [126], which in renal cell carcinoma was found to be associated with MDSCs. Sunitinib increases GM-CSF expression in the TME of renal cell carcinoma, which promotes MDSC survival via a STAT5-dependent pathway. The surviving MDSCs then reduce T cell activity and IFN-γ release to escape immune clearance. In addition, the presence of MDSCs in renal cell carcinoma tissues was correlated with increased expression of proangiogenic factors, suggesting that MDSCs may play a role in antiangiogenic treatment failure [127]. The CD11b^+^Gr1^+^ MDSC population was shown to promote resistance to anti-VEGF treatment in multiple types of refractory tumors, and inhibition of this cell population significantly promoted the outcome of anti-VEGF treatment [128]. Comparison of the actions of sunitinib and bevacizumab suggested that sunitinib, but not bevacizumab, could quickly activate the recruitment of macrophages and MDSCs due to the rapid formation of hypoxic conditions. The combination of bevacizumab and sunitinib abolished the recruitment of CD11b^+^/F4/80^+^/Gr1^-^ myeloid cells and prolonged the survival of GBM patients compared with sunitinib treatment alone [129].

Vatalanib is a VEGFR inhibitor that is selective for VEGFR-2. A study by Achyut et al. suggested that CD68^+^ myeloid cells may be involved in the main non-cell-autonomous mechanism of vatalanib resistance. These myeloid cells exhibit CSF1R^+^ characteristics and can promote angiogenesis and inflammation in the TME of GBM through secreting CXCL7 [130]. The inhibitor of CSF1R could significantly improve vatalanib sensitivity in GBM treatment [131].

#### Other TKIs

Imatinib is a BCR-ABL inhibitor used for the treatment of leukemia. An early study showed that bone marrow cells might protect the CML cell lines K562 and KU812 from imatinib treatment, perhaps due to the activation of Stat5-mediated NF-κB signaling upon an interaction between bone marrow stroma and CML cells [132]. In the K562 cell line, high HO-1 expression in BMSCs was related to imatinib resistance with considerable changes in signaling, including through the PI3K/AKT pathway, Bcl-2 and the CXCL12/CXCR4 axis [133].

Ibrutinib is an inhibitor of Bruton's tyrosine kinase used for the treatment of MCL. The interaction between the TME and lymphoma cells was shown to be mediated by a signaling network centered on the PI3K/AKT pathway. Sustained high levels of AKT phosphorylation ensured stable mTOR signaling, while integrin-β1 increased the TME lymphoma interaction [134]. Another study identified a population of nurse-like cells in the TME of CLL that had a protective effect on ibrutinib-induced tumor cell apoptosis, which could be partially attributed to the inactivation of natural Bcl-2 antagonist in nurse-like cells [135].

## Discussion

As the TME was found to drive significant non-cell-autonomous resistance in multiple types of cancers, treatments that target the TME may regulate the efficacy and effectiveness of antineoplastic drugs; this concept has been well studied and reviewed elsewhere [15, 136], and may provide strategies for new combinations of antineoplastic drugs. For example, locoregional delivery of IL-21 initiated macrophage polarization from the M2 to M1 phenotype, which eliminated immunosuppressive TAMs and induced the T cell response [137]. This type of treatment may be considered as adjuvant therapy to antineoplastic drugs that are susceptible to non-cell-autonomous resistance induced by TAMs. The JAK inhibitor tofacitinib was reported to selectively target the bone marrow microenvironment to block JAK/STAT3 signaling in the stroma [138]. This inhibitory effect works in opposition to BTZ resistance as mentioned above. However, safety and potential side effects of new combinations should be critically evaluated before clinical applications.

Diverse mechanisms by which drugs regulate TME-driven resistance have also been discovered, for instance, studies have found that molecules destroy the TME to improve the release of antineoplastic drugs. Quercetin, a natural compound that blocks the initiation of Wnt16-related signaling in CAFs, can improve the delivery and efficacy of cisplatin [139]. Indeed, recent studies on drug delivery via nanoparticles have shown that drug-containing nanoparticles with supportive components on the surface can destroy the TME of gastric carcinoma and breast cancer, and enhance the efficiency of drug delivery in vitro and in vivo [140, 141]. Nonetheless, it is difficult to predict and judge whether this TME destruction causes harm.

There are several technical difficulties in studying the non-cell-autonomous resistance of cancer cells. Due to tumor heterogenicity, the response to antineoplastic agents may vary among individual tumor cells. The presence of a population of naturally resistant tumor cells makes it difficult to distinguish the non-cell-autonomous and autonomous mechanisms of resistance. Precluding the pre-existence of a resistant population in the tumor will be critical for understanding the role of the TME in acquiring resistance to antineoplastic agents. However, most of the current platforms for studying the drug resistance of tumor cells rely on the in vitro selection of resistant populations, which excludes the influence of other cell types in the TME that may be important in the in vivo acquisition of drug resistance; therefore, these platforms are not suitable for the study of non-cell-autonomous mechanisms of drug resistance. Some attempts to mimic the TME in vitro, such as microenvironment-on-chip, ECM-based tumor cell culture, and tumor-stromal cell coculture, have been made and discussed; however, these systems only partially resemble the actual TME. In vivo selection of resistant populations may identify traits important for non-cell-autonomous drug resistance. A recent study performed in vivo selection of TKI-resistant populations in tumor-bearing mice with acquired resistance to TKI treatment. By isolating cancer cells from resistant tumors in the animal, it was possible to identify whether the resistance was gained by tumor cells themselves or by the influence of the TME [108]. This system could help overcome the current technical problems in studying the non-cell-autonomous mechanism of drug resistance in cancer. In addition, recent studies have attempted to establish human organoids from biopsies to better understand cancer biology and further optimize cancer treatment [142, 143]. Human organoids are cultured ex vivo in 3D, primarily from cancer tissues in individual patients, and therefore retain the signature heterogeneity of the TME, the particular tumor phenotype, and the response to antineoplastic treatment [144]. With these features, human organoids have been proposed as a novel ex vivo tool for estimating the human sensitivity to antineoplastic treatment [145], and they may have broad application in understanding the non-cell-autonomous mechanism of drug resistance in future studies.

## Conclusion

Recent studies have identified TME-driven non-cell-autonomous resistance as a critical mechanism that causes refractoriness of cancers and failure of antineoplastic treatment failure. Factors in the TME, including pH, oxygen supply, immune surveillance, fibroblasts and ECM, can respond to drugs and initiate signalings to activate resistance-associated pathways in tumor cells, such as the AKT, mTOR, NF-κB, and STAT3 pathways. Acquired resistance to particular antineoplastic agents may occur via specific non-cell-autonomous mechanisms, while several non-cell-autonomous mechanisms may together contribute to the resistance of tumor cells towards one particular drug. Although the clinical application of TME-targeting molecules to treat cancer resistance requires additional effort in evaluating efficacy, selectivity, and safety, understanding the involvement of TME-driven non-cell-autonomous resistance may prompt trials of novel combinations of currently available antineoplastic agents.

## Abbreviations

AML: Acute myelogenous leukemia
BC-MSCs: Breast cancer cells with tumor tissue-derived MSCs
BMSC: Bone marrow stromal cell
BTZ: Bortezomib
CAFs: Cancer-associated fibroblasts
CCA: Cholangiocarcinoma
CLL: Chronic lymphocytic leukemia
CML: Chronic myelogenous leukemia
CTX: Cetuximab
ECM: Extracellular matrix
EGFR: Epidermal growth factor receptor
EMT: Epithelial-mesenchymal transition
ERL: Erlotinib
EVs: Vesicles
GBM: Glioblastoma multiforme
GFT: Gefitinib
HA: Hyaluronic acid
HCC: Hepatocellular carcinoma
HER2: Human epidermal growth factor receptor
HIF: Hypoxia-inducible factor
HNSCC: Head and neck squamous cell carcinomas
HSCs: Hepatic stellate cells
IGF: Insulin-like growth factor
IGF1R: Insulin-like growth factor 1 receptor
IR: IGF2-insulin receptor
MDSCs: Myeloid-derived suppressor cells
MGMT: O6-methylguanine-DNA methyltransferase
miRNAs: microRNAs
MM: Multiple myeloma
MSCs: Mesenchymal stem cells
SASP: Senescence-associated secretory phenotype
TAMs: Tumor-associated macrophages
TANs: Tumor-associated neutrophils
TGF-β: Transforming growth factor-β
TME: Tumor microenvironment
TMZ: Temozolomide
VEGF: Vascular endothelial growth factor
